# Astragalus polysaccharides protect against Di-n-butyl phthalate-induced testicular damage by modulating oxidative stress, apoptosis, and the PI3K/Akt/mTOR pathway in rats

**DOI:** 10.3389/fvets.2025.1616186

**Published:** 2025-07-28

**Authors:** Manal R. Bakeer, Seham Samir Soliman, Omaima Ahmed, Fady Sayed Youssef, Ghada E. Ali, Nada H. Aljarba, George D. Zouganelis, Maha M. Rashad

**Affiliations:** ^1^Physiology Department, Faculty of Veterinary Medicine, Cairo University, Giza, Egypt; ^2^Department of Animal Reproduction and Artificial Insemination, Veterinary Research Institute, National Research Centre (NRC), Dokki, Cairo, Egypt; ^3^Department of Cytology & Histology, Faculty of Veterinary Medicine, Cairo University, Giza, Egypt; ^4^Department of Pharmacology, Faculty of Veterinary Medicine, Cairo University, Giza, Egypt; ^5^Biochemistry and Molecular Biology Department, Faculty of Veterinary Medicine, Cairo University, Giza, Egypt; ^6^Department of Biology, College of Science, Princess Nourah bint Abdulrahman University, Riyadh, Saudi Arabia; ^7^School of Sciences, College of Science and Engineering, University of Derby, Derby, United Kingdom

**Keywords:** dibutyl phthalate, astragalus polysaccharides, oxidative stress, reproductive toxicity, apoptosis, PI3K/AKT/mTOR pathway, male infertility

## Abstract

**Introduction:**

Di-n-butyl phthalate (DBP), a common plasticizer, is associated with oxidative stress and male reproductive toxicity. Astragalus polysaccharides (APS) have known antioxidative and anti-inflammatory properties, but their role in male reproductive health has not been fully elucidated.

**Methods:**

Twenty-four male rats were randomly assigned to four groups (*n* = 6 each): control, DBP-only (500 mg/kg/day), APS-only (200 mg/kg/day), and APS + DBP (500 mg/kg/day DBP + 200 mg/kg/day APS). Treatments were administered orally for 8 weeks. Biochemical, histological, and molecular analyses were conducted to evaluate testicular function, oxidative stress markers, and gene expression.

**Results:**

DBP exposure significantly decreased serum testosterone levels, catalase (CAT) activity, lactate dehydrogenase (LDH) activity, and sperm quality, while increasing malondialdehyde (MDA) levels and apoptotic markers *Casp3, Casp9*. APS co-treatment significantly restored antioxidant enzyme activity, improved sperm parameters, reduced MDA levels, and alleviated histopathological damage. Gene expression analysis revealed upregulation of Nrf2 and SOD, and modulation of the PI3K/AKT/mTOR signaling pathway.

**Discussion:**

APS exerts protective effects against DBP-induced testicular damage by enhancing antioxidant defenses and regulating key molecular pathways. These findings highlight the therapeutic potential of APS in preventing male infertility associated with environmental toxicants.

## Graphical abstract

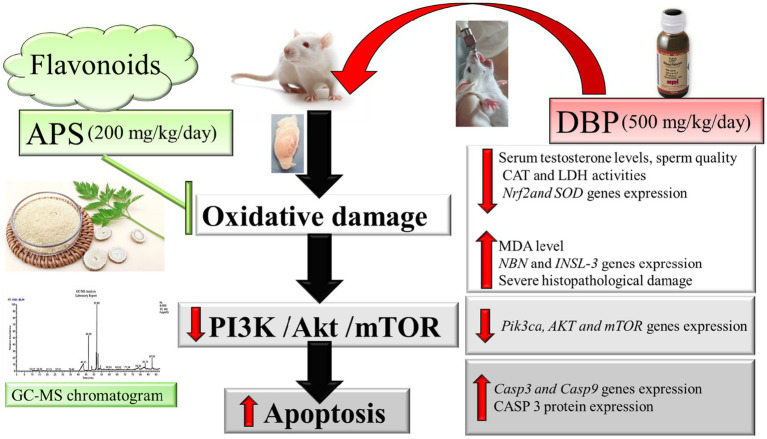

## 1 Introduction

Di-n-butyl phthalate (DBP), a widely used phthalate ester, functions as a plasticizer in numerous consumer products. Extensive research has demonstrated that DBP exposure adversely affects male reproductive health by reducing sperm concentration, motility, and morphology in both animals and humans (1). DBP also disrupts testosterone synthesis and regulation, leading to structural and functional alterations in the testes, including seminiferous tubule degeneration and testicular atrophy (2). Understanding the mechanistic basis of DBP-induced reproductive toxicity is crucial for developing effective therapeutic interventions.

Oxidative stress is a key contributor to DBP-induced testicular damage. The balance between reactive oxygen species (ROS) production and clearance is essential for maintaining testicular integrity (3–5). An excess of ROS disrupts this equilibrium, leading to oxidative damage. Additionally, DBP directly impairs Leydig cell function, resulting in decreased weights of the testis and accessory reproductive organs and reduced testosterone levels (6). Recent studies have suggested that antioxidants may mitigate DBP-induced reproductive dysfunction. For instance, co-administration of naringin, an antioxidant flavonoid, with DBP improved reproductive performance in animal models, highlighting oxidative stress as a central mechanism underlying DBP toxicity (7, 8).

Astragalus polysaccharides (APS), bioactive components derived from Astragalus membranaceus, exhibit potent antioxidant, anti-inflammatory, immune-modulating, and anticancer properties (9). Although APS have been reported to confer protection against oxidative damage in various tissues, their role in preventing DBP-induced testicular toxicity remains unexplored. This study aims to investigate the toxic effects of DBP on the testes and evaluate the protective efficacy of APS against DBP-induced reproductive toxicity. By analyzing physiological, biochemical, pathological, and molecular parameters, this research provides insights into the mechanisms underlying APS-mediated protection and highlights their therapeutic potential in counteracting environmental toxin-induced infertility.

## 2 Materials and methods

### 2.1 Chemicals

DBP (purity ≥98.0%) was procured from Aktin Chemicals, Inc. (Chengdu, China). APS (purity ≥90.0%, Catalog No. A7970) was obtained from Solarbio (Beijing, China). APS was dissolved in deionized water (40 mg/ml) and stored at 4°C, while DBP was emulsified in 0.9% saline.

### 2.2 Characterization of fatty acid composition in *Astragalus membranaceus* using GC–MS analysis

The fatty acid profile of *A. membranaceus* was analyzed using gas chromatography–mass spectrometry (GC–MS). A sample of the total lipid extract (8–12 mg) was transesterified into fatty acid methyl esters (FAMEs). The analysis was performed using a Thermowax 10 capillary column (60.0 m × 0.26 mm i.d., 0.26 μm film thickness; Supelco Inc., Bellefonte, PA, United States) connected to a GC–MS system (PerkinElmer 700 T GC–MS, PerkinElmer, United States). The column temperature was programmed to increase from 140°C to 225°C at a rate of 8°C per minute and held at the final temperature for 23 min. Helium was used as the carrier gas at a constant flow rate of 0.9 mL/min. Fatty acid compounds were identified by comparing their retention times with those of a commercially available standard mixture (Supelco No. 47885-U) and matching the resulting mass spectra with entries in the NIST MS Search 2.1 database. Each compound was quantified by calculating its peak area as a percentage of the total fatty acid content, using the same reference database.

### 2.3 Experimental design and animal model

Twenty-four adult male Wistar albino rats, weighing 180 ± 11 g, were kept in a conventional laboratory setting with unlimited access to food and water, at 22 ± 2°C, 50 ± 5% humidity, and a 12-h light/dark cycle. The animals were acclimated for 7 days prior to the experiment. All procedures were approved by Cairo University’s Institutional Animal Care and Use Committee. The rats were randomly allocated into four experimental groups (*n* = 6 per group) Group 1 (Control). Administered distilled water via oral gavage. Group 2 (DBP only) Administered DBP at a dose of 500 mg/kg/day via oral gavage for 2 months (10). Group 3 (APS only): Administered APS at a dose of 200 mg/kg/day via oral gavage for 2 months (11). Group 4 (APS + DBP) Administered both DBP (500 mg/kg/day) and APS (200 mg/kg/day) under identical conditions.

### 2.4 Sample collection

At the conclusion of the experiment, Isoflurane was used to anesthetize each rat. Blood samples (10 mL per rat) were collected from the femoral artery and centrifuged at 4500 g for 10 min to obtain serum, which was subsequently used for biochemical analyses. Following the collection of blood samples, all rats were humanely euthanized under anesthesia with 10 mg/kg body weight of xylazine and 90 mg/kg body weight of ketamine. The testes were excised. One testis per rat was fixed in 10% neutral-buffered Bouin’s solution for histological assessments, including immunohistochemistry and hematoxylin–eosin (H&E) staining. The remaining testes were stored at −80°C for subsequent real-time PCR analysis.

### 2.5 Semen analysis

Sperm parameters, including count, motility, viability, and morphology, were evaluated following the methodology described in (12). Sperm motility was assessed microscopically within 2 to 4 min after being separated from the cauda epididymis.

### 2.6 Hormone and biochemical assays

A. Hormone Measurement: Serum levels of testosterone were quantified using commercial ELISA kits (DRG, Germany). All assays were performed in duplicate.

B. Biochemical Assays: Testicular tissues were homogenized in 50 mM Tris–HCl buffer (pH 7.4) containing 1.15% potassium chloride. The homogenates were centrifuged at 10,000 g for 15 min at 4°C, and the supernatant was collected for biochemical analyses. Catalase (CAT) activity was measured using hydrogen peroxide as a substrate. Lipid peroxidation was assessed by quantifying malondialdehyde (MDA) levels, expressed as μmol MDA per gram of tissue. Lactate dehydrogenase (LDH) activity was determined using enzymatic assay kits (Spin react, Spain).

### 2.7 Real-time PCR analysis

The relative mRNA expression levels of *Nrf-2, SOD, Casp3, Casp9, NBN, Pik3ca, AKT*, and *mTOR* genes in testicular tissue were quantified using real-time PCR, with *ACTB* as the reference gene. RNA was isolated from approximately 100 mg of testicular tissue using the Total RNA Extraction Kit (Applied Biotechnology, EX02), according to the manufacturer’s instructions. RNA quality and concentration were verified using Nanodrop spectrophotometry at 260 nm and 280 nm (the purity of RNA ranged from 2 to 2.3) (13). Complementary DNA (cDNA) was synthesized using the cDNA Synthesis Kit (Applied Biotechnology, AMP 11) and the SYBR Green PCR Master Mix (Applied Biotechnology, AMP 03) (14). The primer sequences utilized for amplification are listed in Table 1. Each reaction included three biological replicates, analyzed in technical triplicate to ensure reproducibility (15). Template-free negative controls were included in each cycle to prevent contamination. Gene expression levels were quantified using the comparative 2^−ΔΔ^CT method (16).

**Table 1:** Primer sequences used for q RT-PCR.

Gene symbol	Gene description	Reference	Primer sequence
*Nrf 2*	Nuclear factor erythroid 2-like 2	(26)	F: 5′-GGCCCTCAATAGTGCTCAG-3′ R: 5′-TAGGCACCTGTGGCAGATTC-3′
*SOD*	Super oxide dismutase	(27)	F: 5′-GCAGAAGGCAAGCGGTGAAC-3′ R:5′-TAGCAGGACAGCAGATGAGT-3′
*INSL3*	Insulin-like growth factor −3	(19)	F: 5′- GTGGCTGGAGCAACGACA −3′ R: 5′- AGAAGCCTGGTGAGGAAGC -3′
*Casp 3*	Caspase 3	(43)	F: 5′- GGAGCTTGGAACGCGAAGAA −3′ R: 5′- CCATCTCCATCAAAGCCGTG −3′
*Casp 9*	Caspase 9	(44)	F: 5′- AACAACGTGAACTTCTGCCC -3′ R: 5′- ACACAAGCCCATTTCAGGGT −3′
*NBN*	Nibrin gene	(45)	F: 5′- ACCAAGAAGCTGAGCGAGTG −3′ R: 5′- CCAGTTGAAGTTGCCGTCTG −3′
*AKT*	Serine/Threonine Kinase 1	(46)	F: 5′- CTGCCCTTCTACAACCAGGA-3′ R: 5′- GTGCTGCATGATCTCCTTGG −3′
*mTOR*	Mechanistic Target Of Rapamycin Kinase	(46)	F: 5′- TCTGCACTTGTTGTTGCCTC-3′ R: 5′- ACAATCGGGTGAATGATGCG −3′
*Pik3ca*	Phosphatidylinositol-4,5-bisphosphate 3-kinase, catalytic subunit alpha	(46)	F: 5′- TAGTGTCCGGGAAAATGGCT −3′ R: 5′- GGCATGCTCTTCGATCACAG −3′
*ACTB*	Beta-Actin	(24)	F: 5′- TGTCACCAACTGGGACGAT −3′ R: 5′- GGGGTGTTGAAGGTCTCAA −3′

### 2.8 Histopathological examination

Tissue samples were preserved in Bouin’s solution, dried using a series of graded ethanols, cleaned with xylene, and then embedded in paraffin for light microscopy. Sections of 3–4 μm thickness were prepared and subsequently stained with hematoxylin and eosin (H&E) according to the protocol outlined in (17). To evaluate histological alterations, the stained sections were observed under a light microscope.

Immunohistochemistry for Caspase-3: Immunohistochemical analysis was conducted on deparaffinized testicular sections (4-μm thickness) to evaluate apoptosis by detecting caspase-3 expression. The procedure followed the caspase-3 antibody kit (Catalog No. PA5-78921, Thermo Fisher Scientific, United States) as described by Gomaa et al. (18).

Image Analysis of Immunohistochemical Sections: At the Faculty of Dentistry, Cairo University, caspase-3-stained sections were analyzed using Leica QWin 500 digital software (Leica Microsystems, Switzerland). Morphometric analysis was performed to quantify caspase-3 immunostaining as an area percentage. Ten independent fields per group were assessed at ×400 magnification, and positive immunohistochemical staining areas were selected for measurement.

### 2.9 Statistical analysis

SPSS version 27.0 was used to analyze data (IBM, United States). The findings were presented in the form of mean ± SD. Group differences were compared using one-way analysis of variance (ANOVA), and *post-hoc* comparisons were made using Duncan’s test. The significance level was set at *p* < 0.05.

## 3 Results

*In vitro* study.

### 3.1 Gas chromatography–mass spectrometry analysis of the tested polysaccharide-enriched fraction of *Astragalus membranaceus* extract

**Figure 1:**
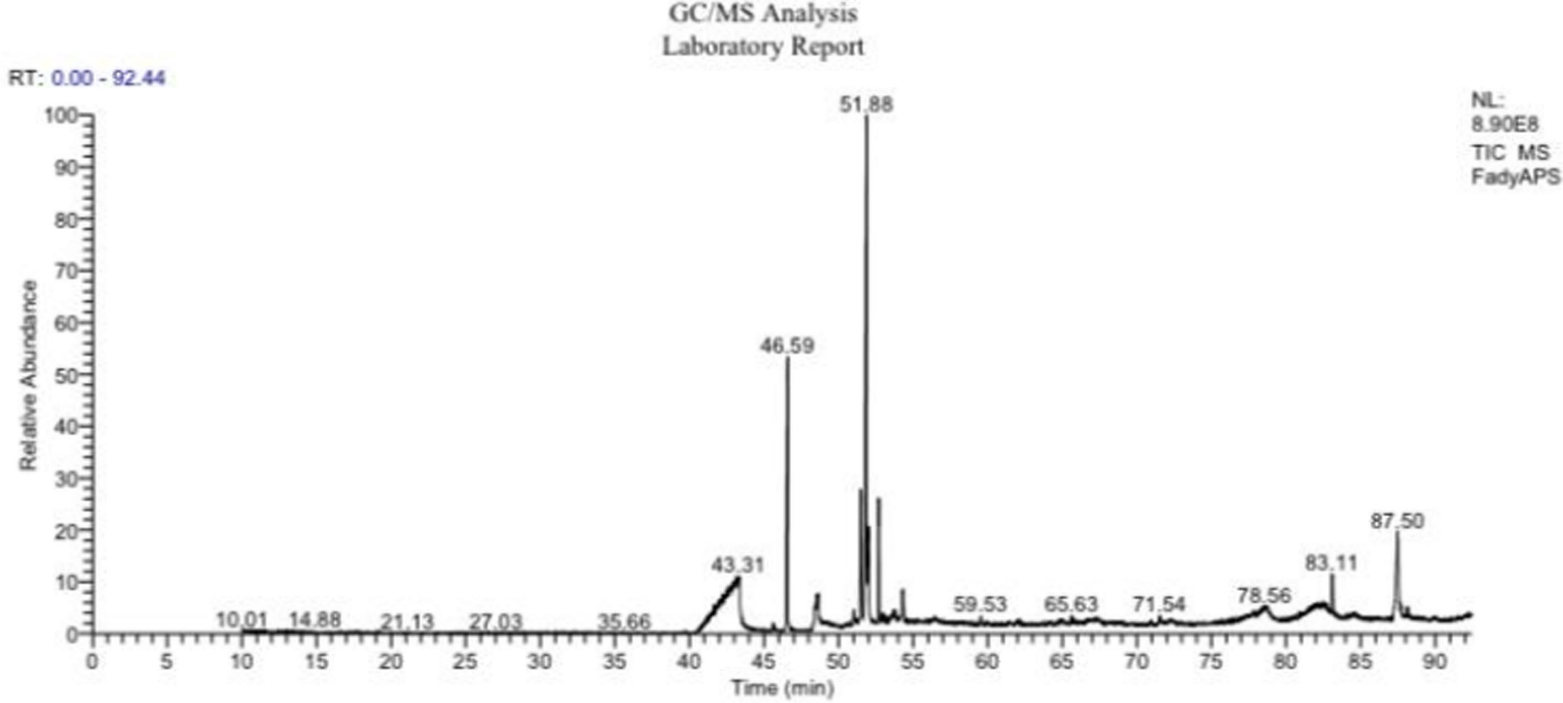
GC–MS chromatogram showing various peaks and their retention times for *Astragalus membranaceus.*

Nineteen different compounds could be detected in *A. membranaceus* via GC–MS separation as shown in Table 2 and Figure 1. A group of five fatty acids, one ketone, and 13 fatty acid esters could be detected in *A. membranaceus.* These compounds were: 2-Heptadecanone (2.15), Hexadecanoic acid, methyl ester (19.74), n-Hexadecanoic acid (4.90), 17-Octadecynoic acid (2.89), 9,12-Octadecadienoic acid (Z, Z)-, methyl ester (2.01), 9-Octadecenoic acid, methyl ester, (E; 33.63), 10-Octadecenoic acid, methyl ester (4.93), Methyl stearate (8.51), 9,12-Octadecadienoic acid, methyl ester, (E, E; 1.82), Oleic Acid (2.06), 9,11-Octadecadienoic acid, methyl ester, (E, E; 2.26), 9-Octadecenoic acid (z; 2.04), Eicosanoic acid, methyl ester (0.54), Docosanoic acid, methyl ester (0.40), 9-Hexadecenoic acid (0.63), 9-Octadecenoic acid (Z)-, tetradecyl ester (0.65), 9-Octadecenoic acid, 1,2,3-propanetriyl ester, (E, E, E; 1.02), Palmitic acid, 2-(tetradecyloxy)ethyl ester (2.76), and Oleic acid, 3-(octadecyloxy)propyl ester (7.06). Two major volatile compounds observed in *A. membranaceus* were 9-Octadecenoic acid, methyl ester, (E) and Hexadecanoic acid, methyl ester.

**Table 2:** GC–MS analysis for various volatile compounds and their corresponding details from *Astragalus membranaceus.*

RT	Compound name	Molecular formula	Molecular weight	Area%	Class
45.63	2-Heptadecanone	C_17_H_34_O	254	2.15	Ketone
46.59	Hexadecanoic acid, methyl ester	C_17_H_34_O_2_	270	19.74	Fatty acid ester
48 0.61	n-Hexadecanoic acid	C_16_H_32_O_2_	256	4.90	Fatty acid
51 0.01	17-Octadecynoic acid	C_18_H_32_O_2_	280	2.89	Fatty acid
51 0.50	9,12-Octadecadienoic acid (Z, Z)-, methyl ester	C_19_H_34_O_2_	294	2.01	Fatty acid ester
51 0.88	9-Octadecenoic acid, methyl ester, (E)	C_19_H_36_O_2_	296	33.63	Fatty acid ester
52 0.00	10-Octadecenoic acid, methyl ester	C_19_H_36_O_2_	296	4.93	Fatty acid ester
52 0.70	Methyl stearate	C_19_H_38_O_2_	298	8.51	Fatty acid ester
53 0.00	9,12-Octadecadienoic acid, methyl ester, (E, E)	C_19_H_34_O_2_	294	1.82	Fatty acid ester
53 0.76	Oleic Acid	C_18_H_34_O_2_	282	2.06	Fatty acid
54 0.31	9,11-Octadecadienoic acid, methyl ester, (E, E)	C_19_H_34_O_2_	294	2.26	Fatty acid ester
56 0.43	9-Octadecenoic acid (z)	C_18_H_34_O_2_	282	2.04	Fatty acid
9 0.54	Eicosanoic acid, methyl ester	C_21_H_42_O_2_	326	0.54	Fatty acid ester
65 0.64	Docosanoic acid, methyl ester	C_23_H_46_O_2_	354	0.40	Fatty acid ester
71 0.54	9-Hexadecenoic acid	C_16_H_30_O_2_	254	0.63	Fatty acid
78 0.55	9-Octadecenoic acid (Z)-, tetradecyl ester	C_32_H_62_O_2_	478	0.65	Fatty acid ester
82 0.53	9-Octadecenoic acid, 1,2,3-propanetriyl ester, (E, E, E)	C_57_H_104_O_6_	884	1.02	Fatty acid ester
83 0.11	Palmitic acid, 2-(tetradecyloxy)ethyl ester	C_32_H_64_O_3_	496	2.76	Fatty acid ester
87 0.50	Oleic acid, 3-(octadecyloxy)propyl ester	C_39_H_76_O_3_	592	7.06	Fatty acid ester

### 3.2 Bioactive compounds and correlation with protective mechanisms

As mentioned in Table 3 several major compounds identified in the polysaccharide-enriched fraction of a. membranaceus via GC-MS exhibit well-established biological activities that are directly relevant to the testicular toxicity induced by DBP. For example, 9-Octadecenoic acid, methyl ester (33.63%) and methyl stearate (8.51%) possess potent anti-inflammatory and lipid-modulating properties, which may help reduce DBP-triggered testicular inflammation and restore lipid homeostasis. Hexadecanoic acid methyl ester and n-Hexadecanoic acid exhibit strong antioxidant and antimicrobial activities which can counteract the oxidative stress and lipid peroxidation caused by DBP exposure. In addition, oleic acid derivatives are known for their neuroprotective and membrane-stabilizing properties, supporting their role in preserving testicular cellular integrity. Importantly, 17-Octadecynoic acid may act as an enzyme inhibitor and has anti-cancer potential, which could contribute to the regulation of apoptotic markers (Casp3, Casp9) and suppression of DBP-induced cell death. These bioactive components may directly counteract DBP-induced oxidative damage, apoptosis, and hormonal disruption—aligning with their modulation of antioxidant systems and cellular signaling pathways (e.g., Nrf2, PI3K/AKT/mTOR) involved in testicular protection.

**Table 3:** Major bioactive compounds identified in polysaccharide-enriched fraction of *A. membranaceus* by GC–MS.

Compound name	Area (%)	Compound type
9-Octadecenoic acid, methyl ester, (E)	33.63	Fatty acid ester
Hexadecanoic acid, methyl ester	19.74	Fatty acid ester
Methyl stearate	8.51	Fatty acid ester
Oleic acid, 3-(octadecyloxy)propyl ester	7.06	Fatty acid ester
n-Hexadecanoic acid	4.9	Fatty acid
Palmitic acid, 2-(tetradecyloxy)ethyl ester	2.76	Fatty acid ester
17-Octadecynoic acid	2.89	Fatty acid

### 3.3 Antioxidant activity of polysaccharide-enriched fraction of *Astragalus membranaceus in vitro*

**Figure 2:**
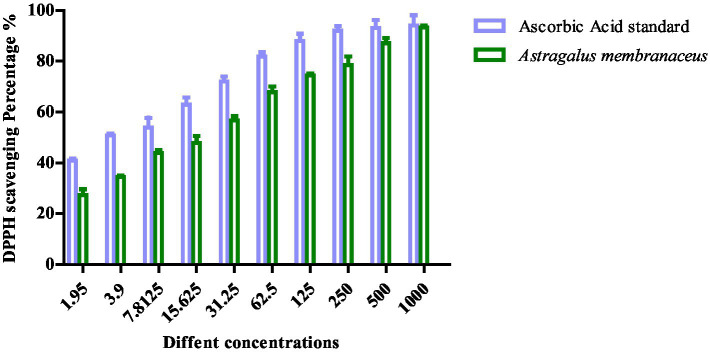
The percentage of free radical scavenging activity of APS with DPPH against that of ascorbic acid.

The graph illustrates the DPPH scavenging activity (%) of *Astragalus membranaceus* extract compared to the ascorbic acid standard across a range of concentrations (1.95–1,000 μg/mL). As the concentration increases, both samples show a corresponding increase in antioxidant activity. However, ascorbic acid consistently exhibits higher DPPH scavenging percentages than *Astragalus membranaceus* at all tested concentrations, indicating stronger antioxidant potential. Notably, at higher concentrations (500 and 1,000 μg/mL), the antioxidant activities of both samples converge, suggesting that *Astragalus membranaceus* possesses significant antioxidant properties, especially at higher doses. The DPPH scavenging assay for *A. membranaceus* showed its notable antioxidant activity with IC_50_ = 14.72 ± 0.71 μg/mL relative to ascorbic acid standard, which had IC_50_ = 2.99 ± 0.71 μg/mL as depicted in Figure 2.

### 3.4 Testosterone levels

**Figure 3:**
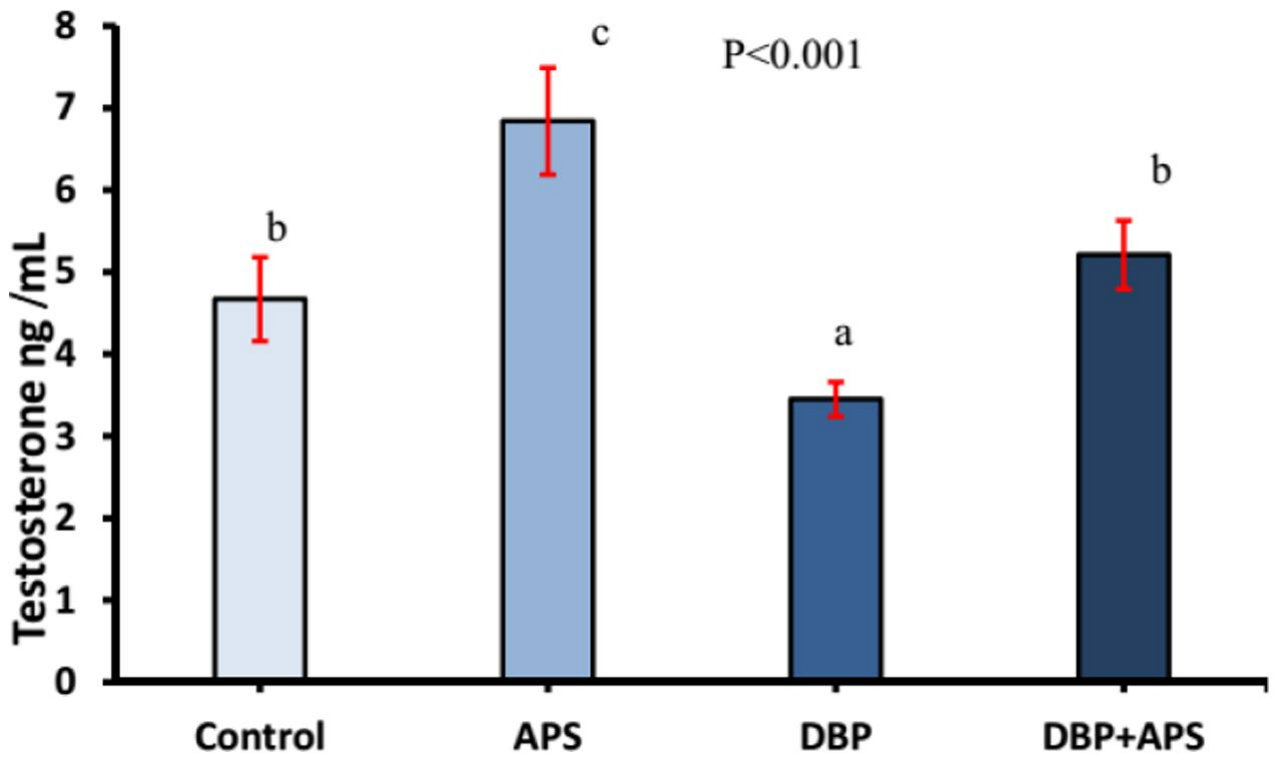
Mean serum testosterone concentrations (ng/ml) in adult male albino rats treated with DBP and APS with error bars. Different superscript letters (a,b,c) indicate significance at *p* < 0.05.

As shown in Figure 3, serum testosterone levels decreased (*p* < 0.001) in the DBP-treated group compared to the control group. However, co-treatment with APS restored (*p* < 0.001) testosterone levels in the DBP + APS group, which showed significant improvement compared to the DBP group.

### 3.5 Semen analysis

**Figure 4:**
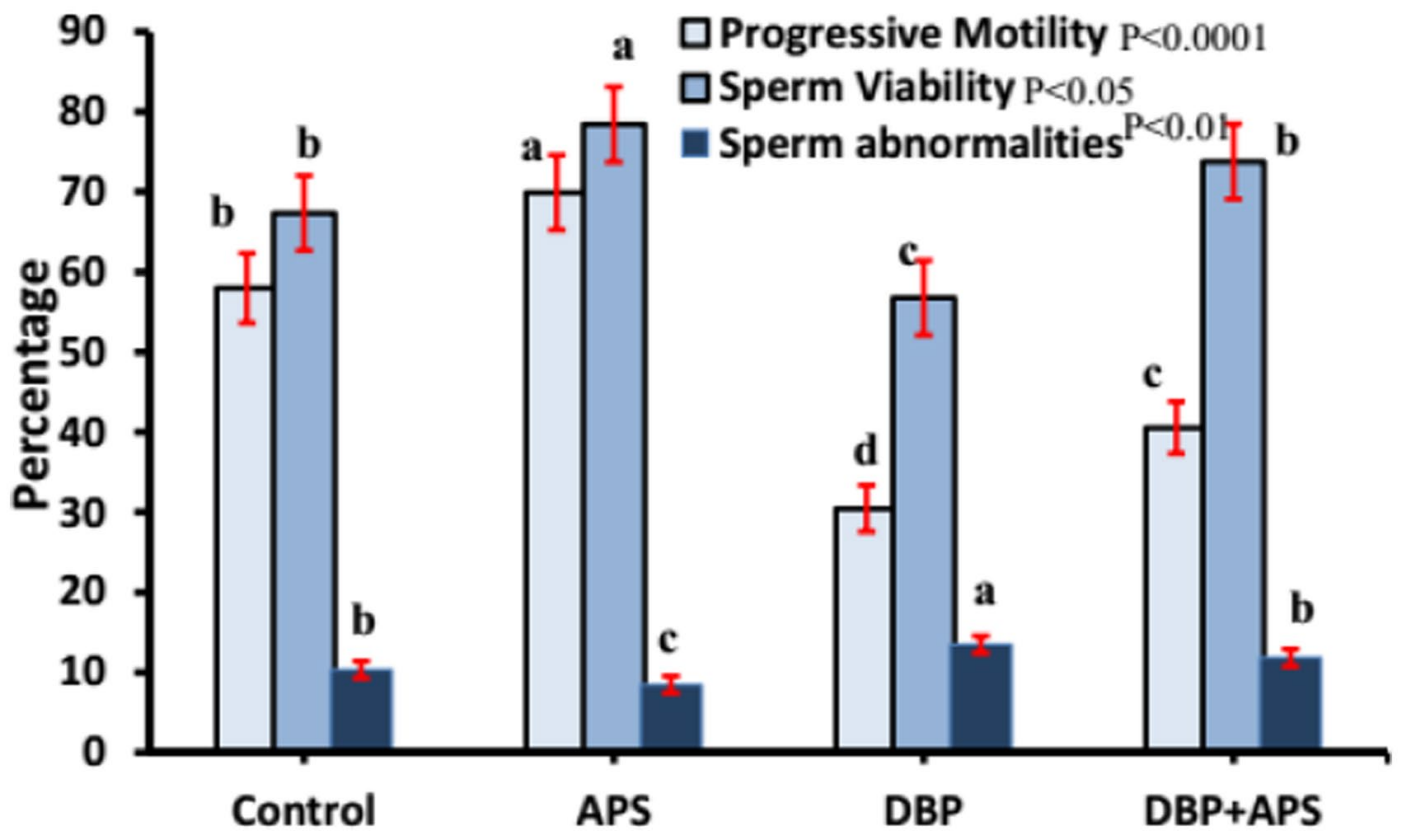
Mean percentage of sperm motility, viability, and abnormality in adult male albino rats treated with DBP and APS with error bars. Different superscript letters (a, b, c, d) indicate significance at p < 0.05.

**Figure 5:**
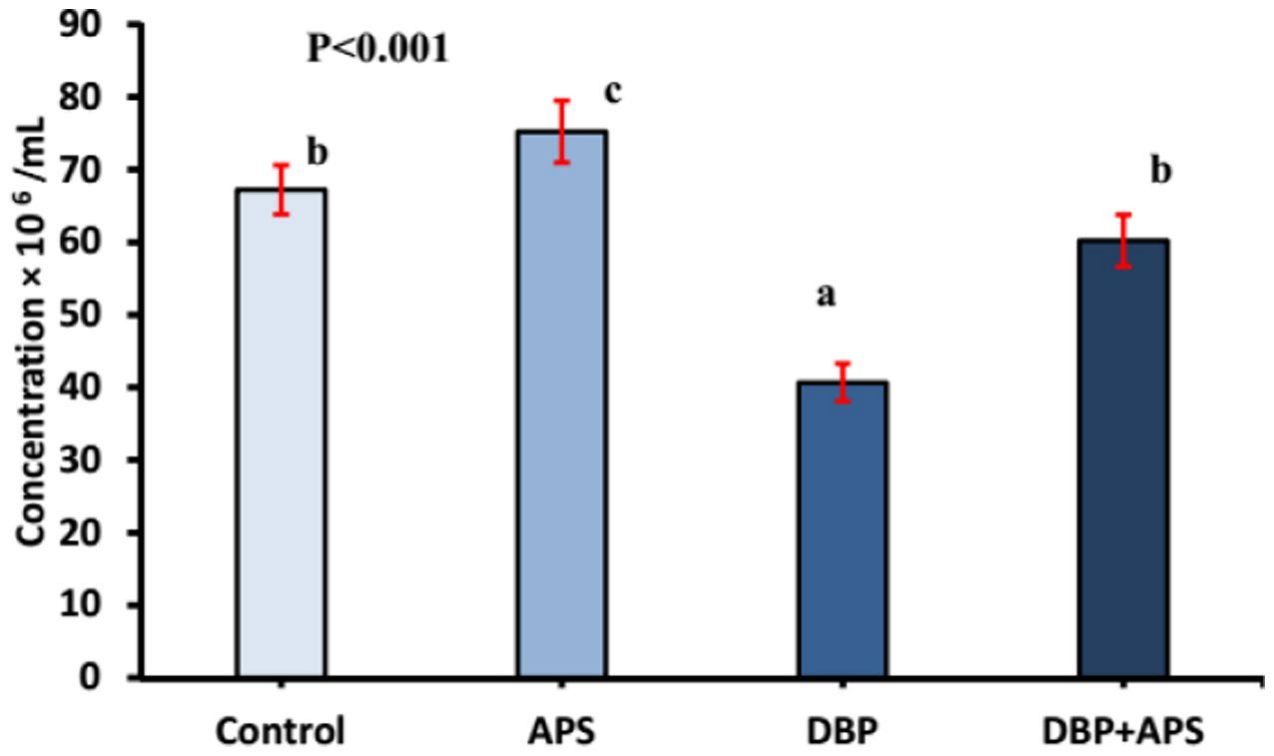
Mean sperm concentration (x10^6^/ml) in adult male albino rats treated with DBP and APS with error bars. Different superscript letters (a, b, c) indicate significance at p < 0.05.

Sperm motility (*p* < 0.0001) and viability (*p* < 0.05; Figure 4) and concentration (Figure 5; *p* < 0.001) of the DBP group exhibited significant reductions compared to the control group (*p* < 0.05). Conversely, DBP treatment increased (*p* < 0.01) sperm abnormalities (Figure 4). Co-treatment with APS ameliorated these effects, resulting in significant improvements in sperm motility, viability, and concentration as well as reduced sperm abnormalities compared to the DBP group.

### 3.6 Testicular enzyme activity

**Figure 6:**
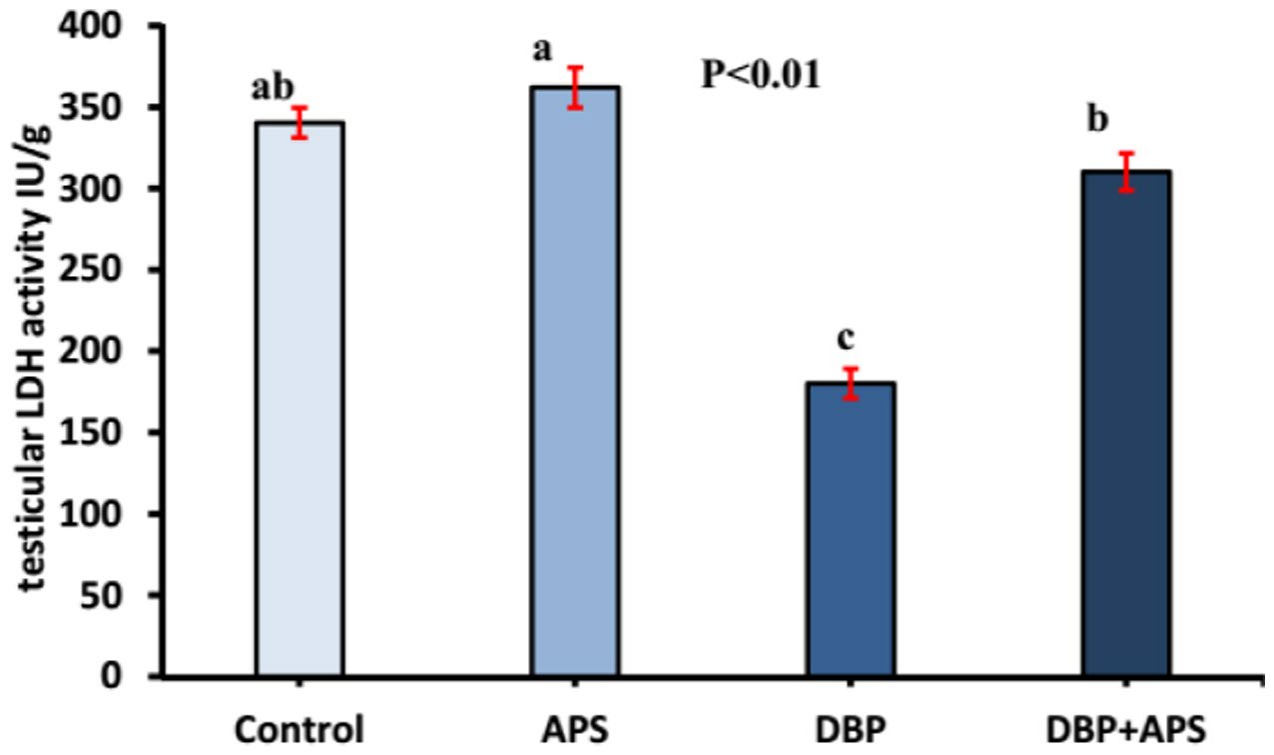
Mean testicular lactate dehydrogenase activity (LDH IU/g) in adult male albino rats treated with DBP and APS with error bars. Different superscript letters (a,b,c) indicate significance at p < 0.05.

The testicular LDH levels in the DBP-treated group were reduced (*p* < 0.01) compared to the control group (Figure 6). APS co-treatment significantly increased LDH levels in the DBP + APS group compared to the DBP group.

### 3.7 Oxidative stress index in testicular tissue

**Figure 7:**
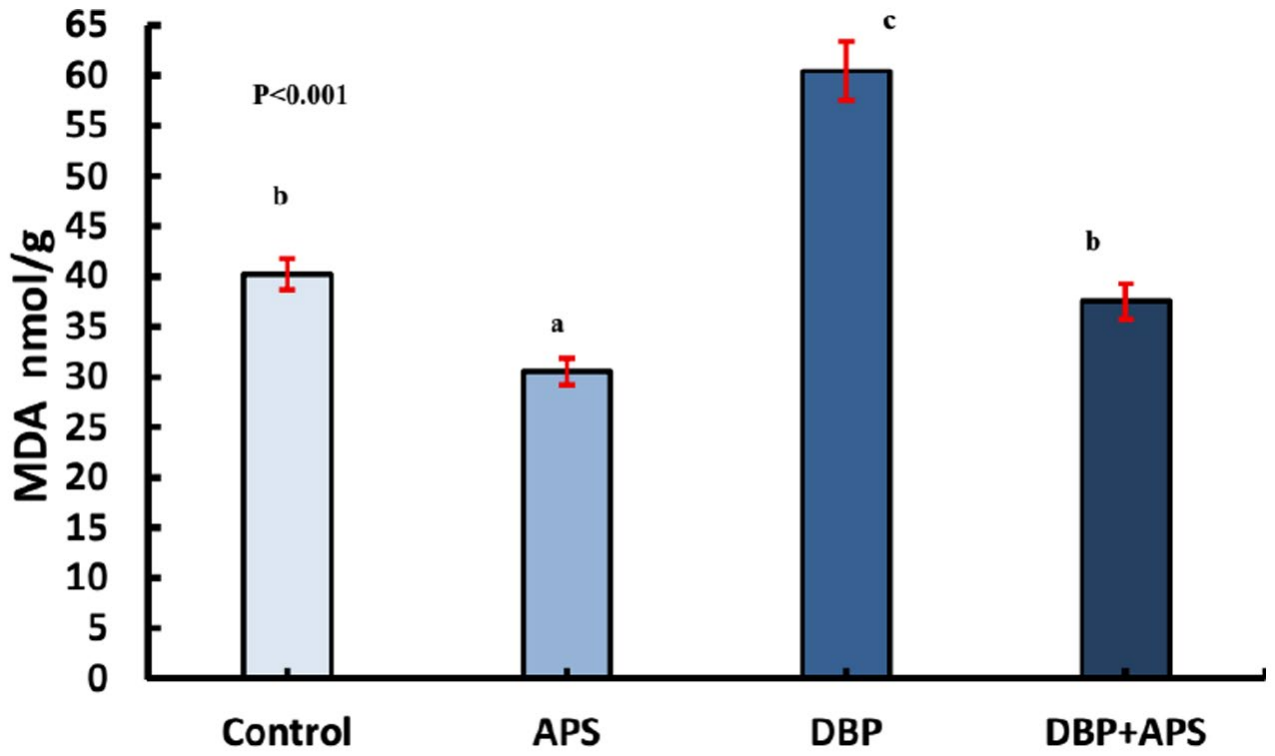
Mean testicular malondialdehyde levels (MDA nmol/g) in adult male albino rats treated with DBP and APS with error bars. Different superscript letters (a, b, c) indicate significance at p < 0.05.

**Figure 8:**
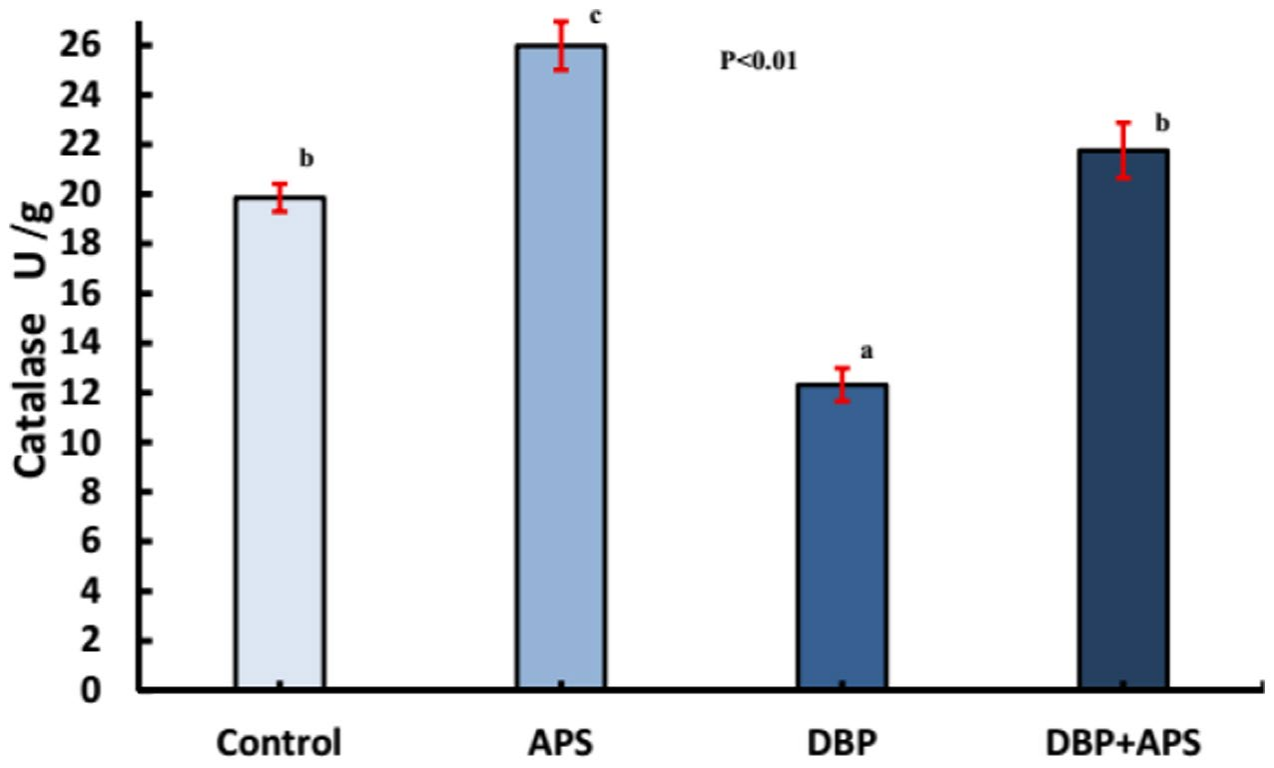
Mean testicular Catalase activity (U/g) in adult male albino rats treated with DBP and APS with error bars. Different superscript letters (a, b, c) indicate significance at p < 0.05.

DBP treatment increased MDA (*p* < 0.05; Figure 7) but decreased catalase (CAT; *p* < 0.05; Figure 8) in testicular tissue compared to the control group. APS co-treatment reversed these changes, significantly reducing MDA levels and enhancing CAT activity in the DBP + APS group compared to the DBP group.

### 3.8 mRNA expression of the *INSL3* gene

**Figure 9:**
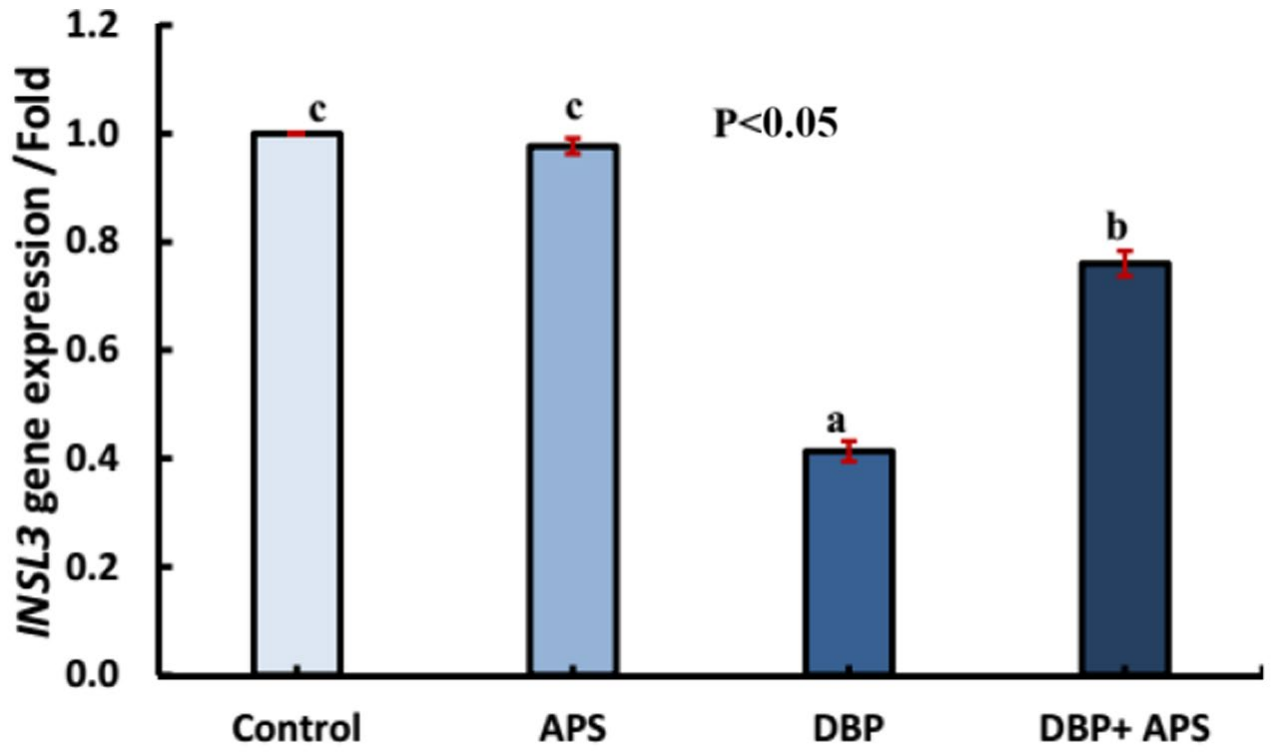
mRNA expression of the *INSL3* gene in testicular tissue of adult male albino rats treated with DBP and APS with error bars. Different superscript letters (a, b, c) indicate significance at p < 0.05.

The oral administration of DBP downregulated (*p* < 0.05) the mRNA expression of the *INSL3* gene compared with the control group. However, co-treatment with APS upregulated (*p* < 0.05) *INSL3* expression relative to the DBP group, as shown in Figure 9.

### 3.9 mRNA expression of antioxidant-related genes (*Nrf-2* and *SOD*)

**Figure 10:**
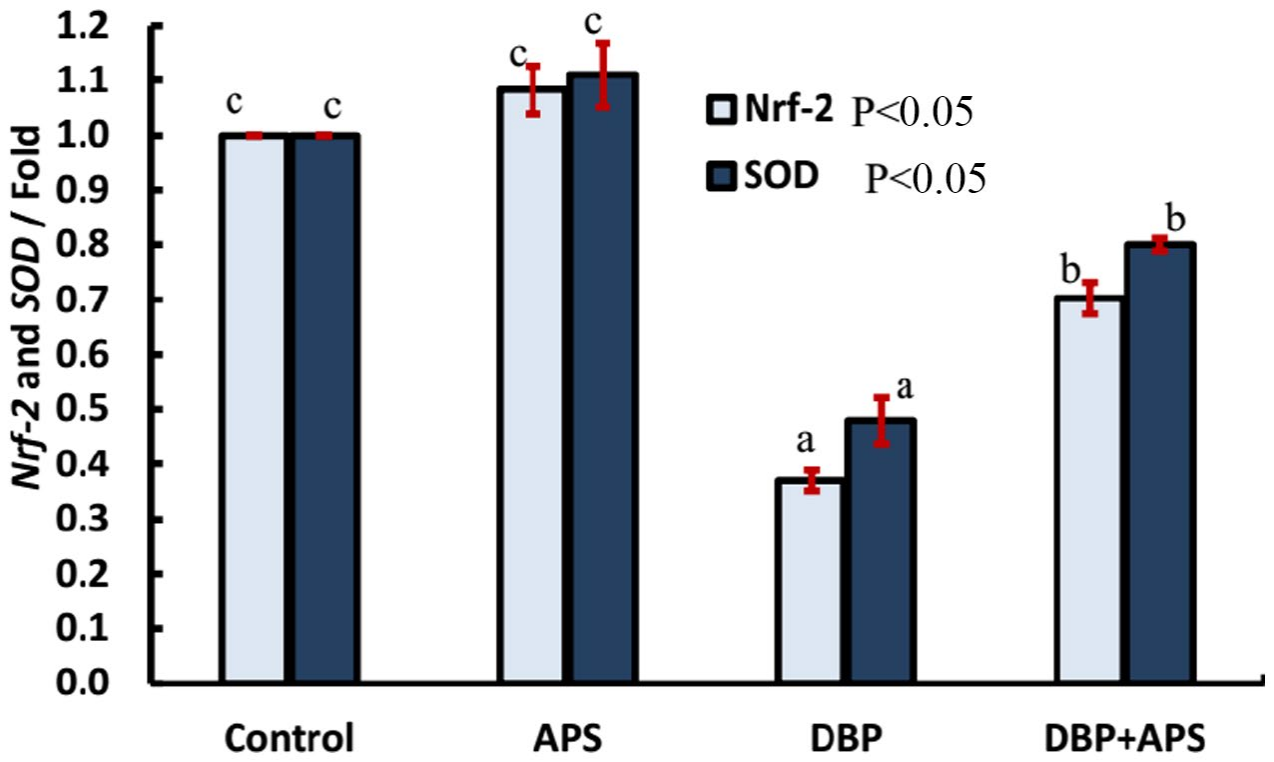
mRNA expression of antioxidant-related genes: *Nrf-2* and *SOD* in testicular tissue of adult male albino rats treated with DBP and APS with error bars. Different superscript letters (a, b, c) indicate significance at p < 0.05.

The DBP-treated group exhibited a down-regulation of *Nrf-2* (*p* < 0.05) and *SOD* (*p* < 0.05) gene expression compared to the control group. Conversely, APS co-treatment significantly upregulated the expression of these genes relative to the DBP group, as illustrated in Figure 10.

### 3.10 mRNA expression of the DNA damage-responsive gene (*NBN*)

**Figure 11:**
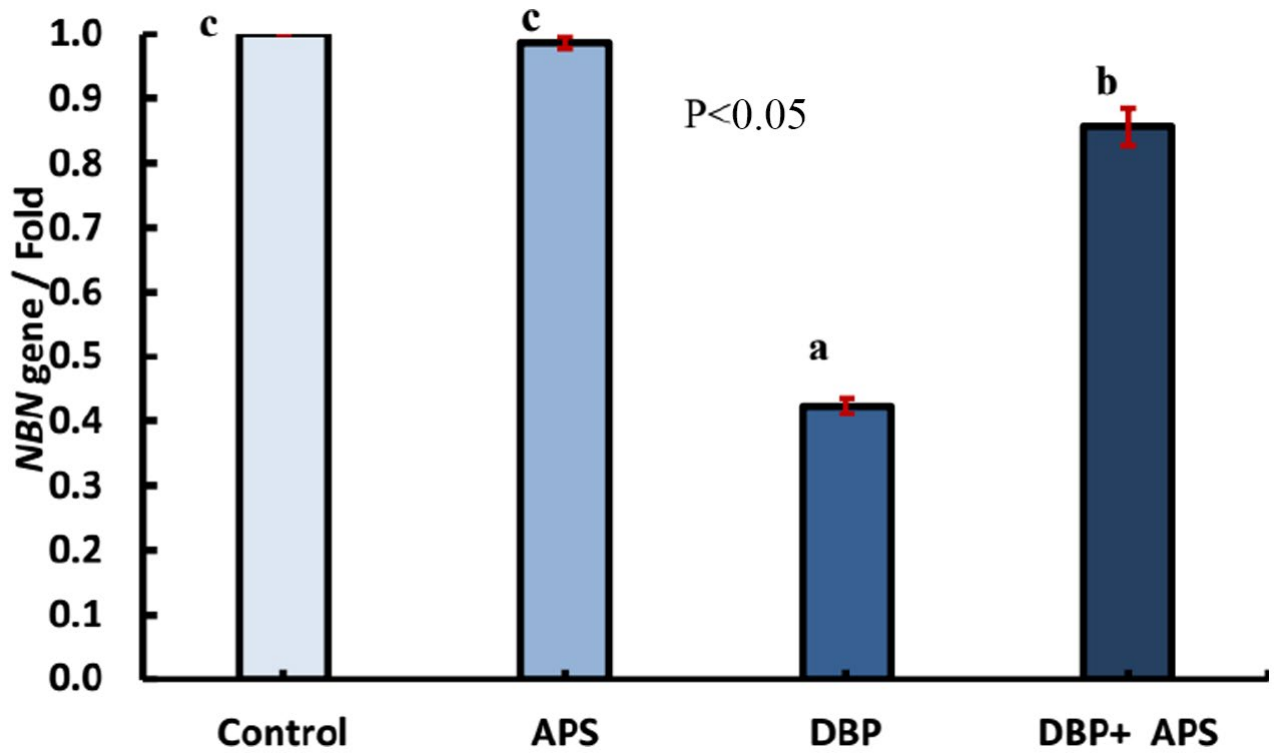
mRNA expression of the *NBN* gene in testicular tissue of adult male albino rats treated with DBP and APS with error bars. Different superscript letters (a,b,c) indicate significance at p < 0.05.

Oral exposure to DBP upregulated (*p* < 0.05) the expression of the *NBN* gene compared to the control group. In contrast, co-treatment with APS downregulated (*p* < 0.05) NBN expression compared to the DBP group, as depicted in Figure 11.

### 3.11 mRNA expression of apoptosis-related genes (*Caspase-3* and *Caspase-9*)

**Figure 12:**
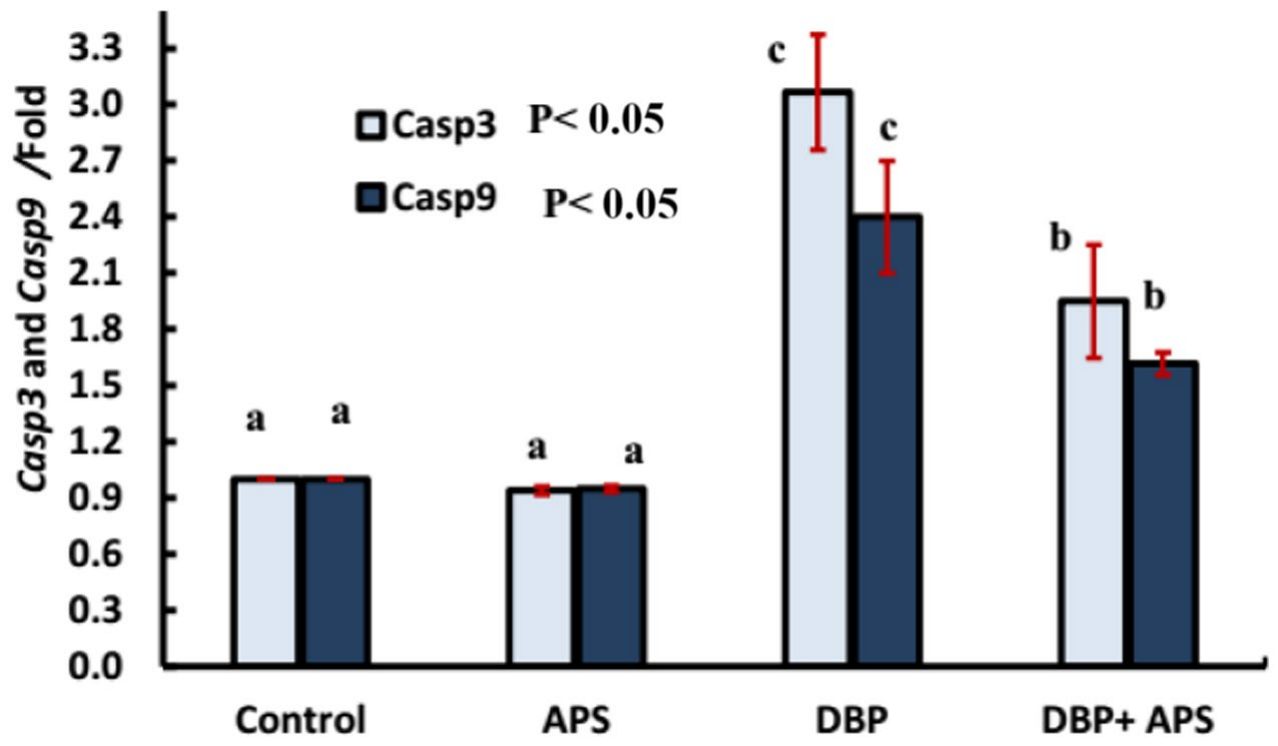
mRNA expression of apoptosis-related genes: *Casp3* and *Casp9* genes in testicular tissue of adult male albino rats treated with DBP and APS with error bars. Different superscript letters (a, b, c) indicate significance at p < 0.05.

The DBP-treated group demonstrated upregulation of *Caspase-3* (*Casp3*; *p* < 0.05) and *Caspase-9* (*Casp9*; *p* < 0.05) gene expression compared to the control group. Co-treatment with APS significantly downregulated the expression of both genes compared to the DBP-treated group, as shown in Figure 12.

### 3.12 mRNA expression of *PI3K/Akt/mTOR* signaling pathway-related genes (*PI3K*, *Akt*, and mTOR)

**Figure 13:**
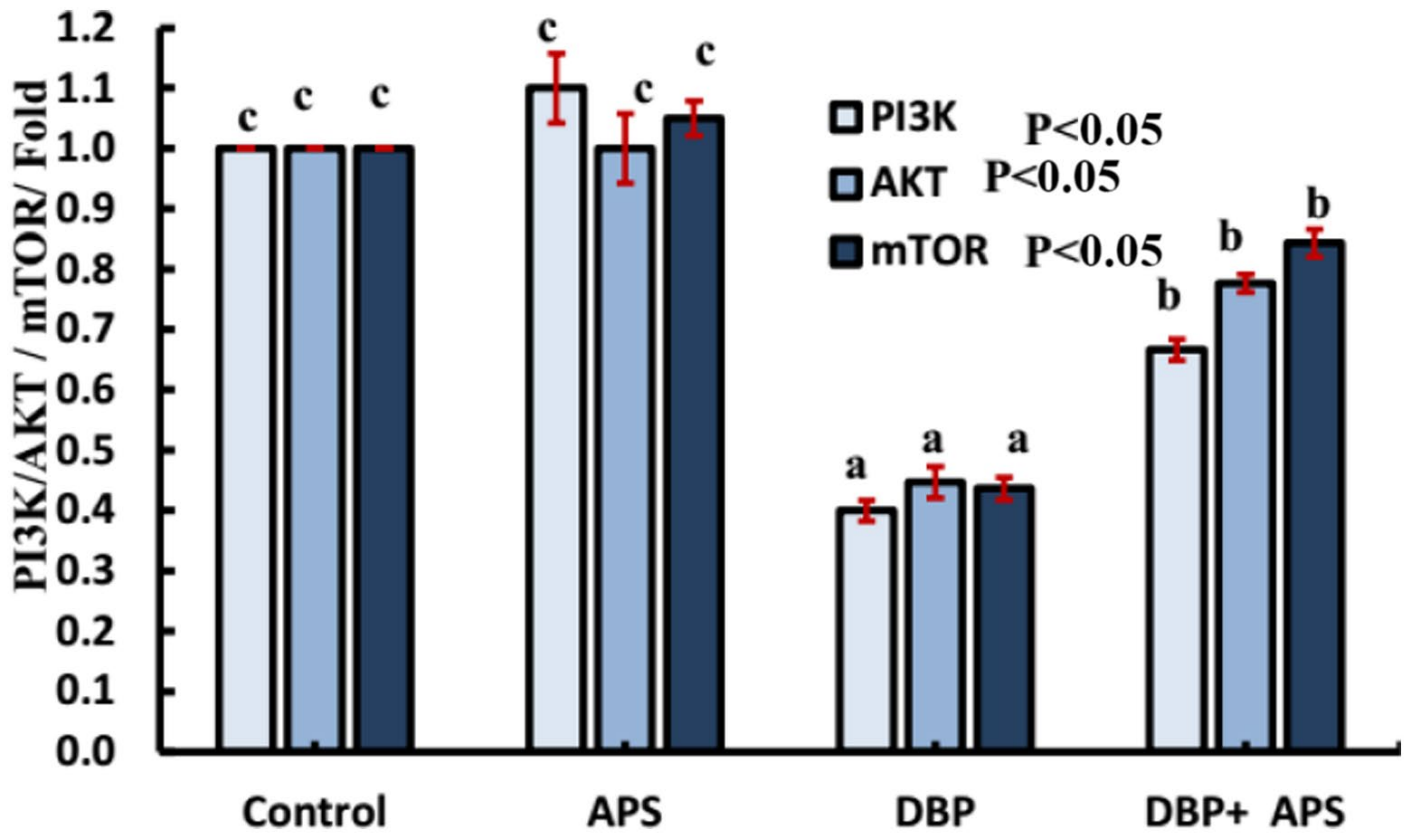
mRNA expression of PI3K/AKT signaling-related *Casp9* genes in testicular tissue of adult male albino rats treated with DBP and APS with error bars. Different superscript letters (a, b, c) indicate significance at p < 0.05.

The expression levels of *Pik3ca, AKT*, and *mTOR* genes were down-regulated (*p* < 0.05) in the DBP-treated group compared to the control group. APS co-treatment reversed (*p* < 0.05) this effect, resulting in up-regulation of these genes relative to the DBP-treated group, as illustrated in Figure 13.

### 3.13 Histopathological observation

**Figure 14:**
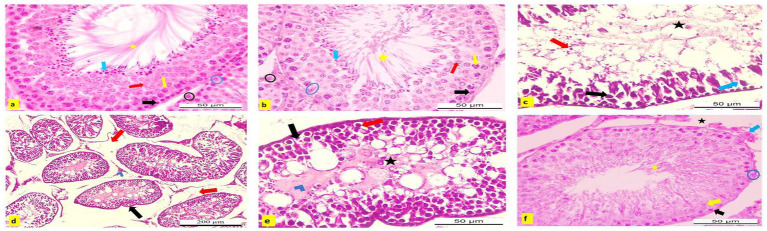
**(a–f)** Hematoxylin and eosin (H&E) stained testicular sections from adult male albino rats. **(a)** Control and **(b)** APS group showing normal active seminiferous tubule with spermatogenic cells; spermatogonia (black arrow), primary spermatocyte (yellow arrow), secondary spermatocyte (red arrow), spermatid (blue arrow), Sertoli cells (blue circle) with lumen filled with sperms (yellow star). Normal interstitial tissue containing Leydig cells (blue circle; X400). Testicular sections from DBP group **(c)** showed degeneration, necrosis (blue arrow), and pyknosis of spermatogenic cells with sloughing into the lumen (red arrow), which appeared devoid of sperm (black star). Germinal epithelial cells could not be differentiated within the wall (black arrow). **(d)** Degenerated seminiferous tubules appeared irregular in shape (black arrow) with widened interstitial spaces (edema; red arrow) and necrosis of Leydig cells (blue chevron; X400). **(e)** Spermatogenic cells displayed ballooning and vacuolization (red arrow), degeneration (black arrow) with necrotic debris in the tubular lumina (blue chevron), and absence of sperms (black star; X100). **(f)** DBP + APS group exhibited some nearly normal seminiferous tubules with several layers of spermatogenic cells, spermatogonia (black arrow), primary spermatocytes (yellow arrow), with Sertoli cell (blue circle), and the lumen contained sperms (yellow star). Narrow interstitial tissue, no edematous area (black star), and nearly normal Leydig cells (blue arrow; X400).

Microscopic analysis of H&E-stained testicular tissues from adult male albino rats in the negative control and APS-exposed groups revealed a typical testicular structure. The seminiferous tubules were intact, rounded, and functionally active, with a normal germinal epithelium composed of Sertoli cells characterized by large, oval, vesicular nuclei. The tubules were separated by narrow interstitial spaces containing healthy Leydig cells. The germinal epithelium exhibited a full spectrum of spermatogenic cells, including spermatogonia, primary and secondary spermatocytes, and spermatids, with spermatozoa filling the tubular lumina (Figures 14a,b).

Conversely, testicular sections from DBP-exposed rats showed severe histopathological damage compared to the control and APS groups. Notable alterations included degeneration, necrosis, and pyknosis of spermatogenic cells, along with their detachment into luminal spaces that lacked sperm. The germinal epithelium and Sertoli cells appeared indistinct, while seminiferous tubules were irregular and surrounded by widened interstitial spaces due to edema. Additionally, Leydig cells exhibited necrosis (Figure 14d). Spermatogenic cells showed ballooning, cytoplasmic vacuolization, degeneration, apoptosis, and accumulation of necrotic debris within the tubular lumina, accompanied by an absence of sperm (Figures 14c,e).

However, testicular sections from the DBP + APS co-treated group showed considerable improvement. Some seminiferous tubules appeared nearly normal, with reduced degeneration and necrosis compared to the DBP group. These tubules contained multiple layers of spermatogenic cells, Sertoli cells with clear vesicular nuclei, and lumina filled with sperm. The interstitial tissue was narrow and free from edema, while Leydig cells appeared nearly normal (Figure 14f).

### 3.14 Immunohistochemical observations

**Figure 15:**
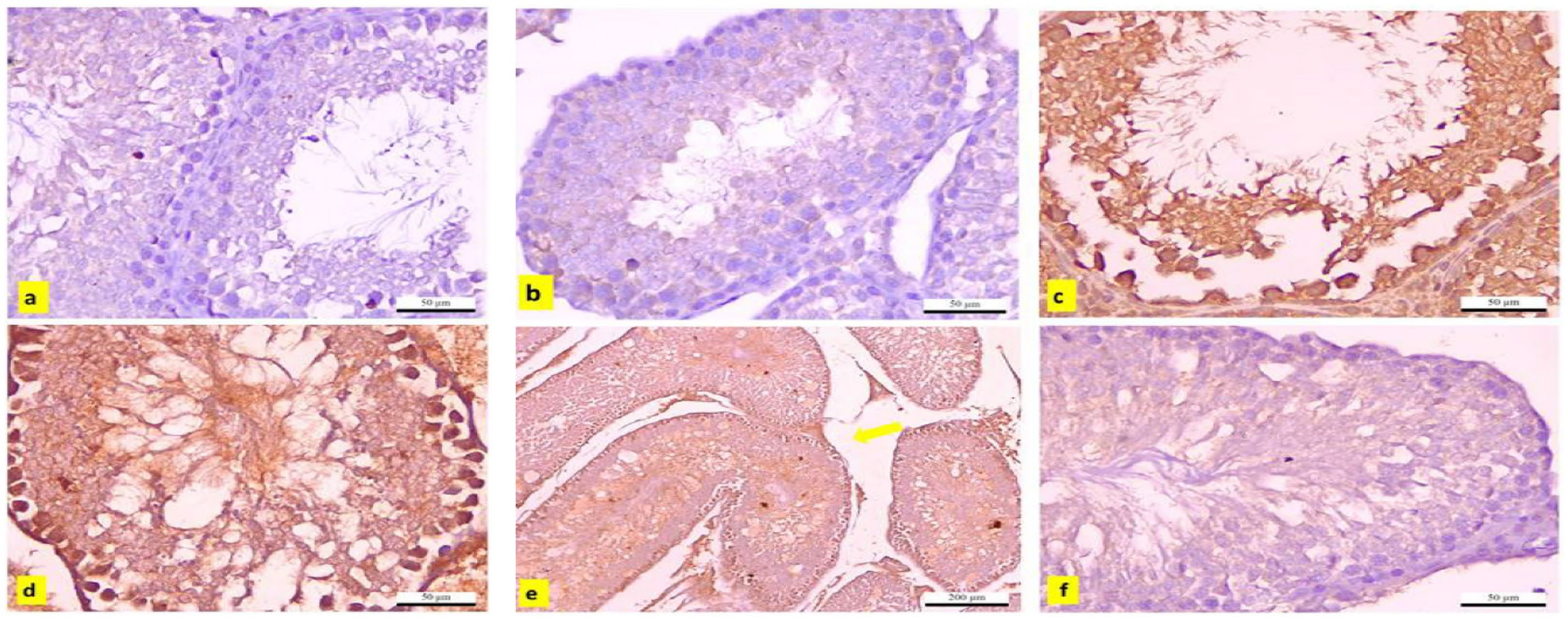
**(a–f)** Immunohistochemically caspase-3-stained testicular sections from adult male albino rats. **(a)** Control and **(b)** APS group exhibiting negative immune reactivity to caspase-3 (x400), DBP-exposed group **(c–e)** shows strong positive immunoreaction(x400). **(e)** The interstitial tissue edema (yellow arrow; x100). **(f)** DBP+APS group shows a negligible immune reaction with several layers of spermatogenic cells (x400).

The testicular tissues from the control and APS groups exhibited negligible immunoreactivity to caspase-3 (Figures 15a,b). In contrast, tissues from the DBP group showed strong positive immunoreactivity (Figures 15c,d). Additionally, the interstitial tissue in the DBP group was markedly affected by edema (Figure 15e). However, the APS co-treated group displayed moderate caspase-3 immunoreactivity (Figure 15f). Quantitative analysis revealed a significant increase (*p* < 0.05) in the percentage area of caspase-3-positive immunoreactive cells in the DBP group, with a 19.876-fold increase compared to the control and APS groups. On the other hand, the DBP + APS co-treatment group demonstrated a significant reduction (*p* ≤ 0.05) in caspase-3-positive area percentage, showing a 7.589-fold decrease relative to the DBP-only group (Table 4).

**Table 4:** The area% covered by caspase3 positive immunoreactive cells in the testicular tissue.

Experimental groups	Area % (Mean ± SEM)
NC	0.0 ± 0^b^
APS	0.0 ± 0^b^
DBP	19.876 ± 0.806^a^
DBP + APS	7.589 ± 0.947^b^

C, Control group; APS, Astragalus polysaccharides (200 mg/Kg); DBP, Di-n-butyl phthalate (500 mg/kg).

## 4 Discussion

Dibutyl phthalates (DBP) are well-documented toxicants that cause reproductive and developmental harm in animals (7). APS have a potential medical impact, although its effects on the reproductive system have received little attention. Thus, the present study investigated the mechanisms underlying DBP-induced male reproductive toxicity and evaluated the protective effects of APS.

Our results indicated that DBP induced significant testicular damage associated with oxidative stress and apoptosis via the PI3K/Akt/mTOR signaling pathway. In the current investigation, significant oxidative stress in testicular tissue was linked to reduced testicular weight, sperm abnormalities, testicular injury, suppression of Leydig cell steroidogenesis, and disruption of oxidative stress biomarkers. Consistent with previous findings, DBP exposure led to a significant increase in abnormal sperm morphology and adversely affected sperm parameters, including concentration and motility, likely due to testicular atrophy and spermatogenic arrest (19–21).

The testicular injury was confirmed by the significant decrease in LDH activity and the severe histopathological damage, which included degeneration, necrosis, and pyknosis of spermatogenic cells, as well as impaired spermatogenesis due to disrupted germinal epithelium. These findings are consistent with those of (22). In addition to our earlier histological findings, the decreased testosterone level and the downregulation of INSL3 mRNA gene expression further indicated Leydig cell injury. A key mechanism of DBP toxicity appears to be the suppression of testosterone production, which is controlled by the hypothalamus-pituitary-testis (HPT) axis and is fundamental for spermatogenesis (23). Our results demonstrated that DBP exposure disrupted the HPT axis, as indicated by decreased serum testosterone levels. INSL3 is a reliable biomarker of Leydig cell activity and dysfunction. This observation is consistent with an earlier study, which reported that prenatal DBP exposure significantly downregulated *INSL3* gene expression, underscoring DBP’s detrimental effects on Leydig cells (14). Furthermore, elevated MDA levels, diminished CAT activity, and downregulation of *Nrf2* and *SOD* gene expression highlight the role of DBP in inducing oxidative stress. Excessive ROS exacerbates lipid peroxidation, compromises sperm membrane integrity, and impairs motility and morphology, as previously documented by Higuchi et al. (24). *Nrf2*, a key regulator of cellular redox homeostasis, governs the synthesis of detoxification and antioxidant enzymes (25). CAT and SOD are essential antioxidant enzymes that prevent oxidative damage to cellular macromolecules, including proteins, lipids, and DNA. DNA damage triggers the expression of *NBN*. *NBN* is a DNA damage response gene that promotes genomic stability, double-strand break repair, and tissue regeneration (26, 27). Oxidative stress induces downregulation of PI3K/Akt/mTOR signaling through a signal transduction cascade. PI3K catalyzes the phosphorylation and translocation of Akt, which controls the activation of mTOR (28). mTOR is a key regulator that modulates numerous pathways, including apoptosis (29). It induces apoptosis by stimulating the inositol-requiring protein-1/c-Jun N-terminal kinase (IRE1/JNK) pathway, which activates caspase-3 and inhibits the expression of the anti-apoptotic *Bcl-2* (30). Our findings revealed that DBP exposure significantly downregulated the expression of *Pik3ca*, *AKT*, and *mTOR* genes and enhanced the apoptotic process, as evidenced by elevated expression of *Casp3* and *Casp9* genes and a strong caspase-3 response in the immunohistochemical analysis. These results are consistent with studies conducted by Chen et al. (31), Li et al. (32), and Wang et al. (33), which demonstrated that DBP inhibited the PI3K/Akt/mTOR pathway and induced apoptosis in rat models and pig testicular cells.

Natural medicines are gaining prominence in treating various disorders due to their comparatively fewer adverse effects than synthetic pharmaceuticals, which are often linked to significant public health risks, including severe side effects and the development of drug resistance. Herbal remedies—especially those derived from medicinal plants with bioactive properties such as antibacterial, antioxidant, antidiabetic, antiparasitic, and anticancer activities—are receiving increased attention for their use in human, veterinary, and poultry health (34–36). Recently, APS has attracted great worldwide attention owing to its multiple biological activities. Besides its high content of polysaccharides, APS is also rich in flavonoid compounds, saponin compounds, alkaloids, volatile fatty acids, and fatty acid esters, which possess diverse pharmacological properties, including antioxidant, immunomodulatory, antitumor, antidiabetic, antiviral, hepatoprotective, anti-inflammatory, and neuroprotective activities (9). In the present study, APS was able to ameliorate DBP-induced testicular damage and oxidative stress, as evidenced by the restoration of testicular weight, reduction in sperm abnormalities, improvement in testicular architecture, enhancement of germinal epithelial thickness, and reduction in degeneration and necrosis. Additionally, APS improved Leydig cell function and oxidative stress biomarkers. Notably, APS modulated the expression of genes related to the PI3K/Akt/mTOR pathway and apoptosis, as well as the immunohistochemical expression of caspase-3. Our findings align with a recent study that linked improved sperm characteristics, antioxidant capacity, and reproductive performance in rams to the administration of APS and its nanosized form (37). An earlier study by Hu (38) highlighted APS’s protective effects against oxidative damage caused by bisphenol A (BPA), a known disruptor of male fertility. APS supplementation improved sperm motility by reducing MDA levels while upregulating CAT and SOD activities. Additionally, APS enhanced mitochondrial membrane potential and energy production while preserving tyrosine phosphorylation proteins essential for sperm flagellar function. Moreover, Zhang et al. (39), in their *in vitro* study on preserved dairy goat sperm, attributed the cryoprotective role of APS to its strong antioxidant and antiapoptotic effects, as well as its modulation of sperm energy metabolism. In 2021, Wang and his colleagues also found that the mitochondrial integrity of frozen bull semen was improved, and MDA and ROS levels were reduced after APS addition (23).

The DPPH radical scavenging assay serves as a well-established, rapid *in vitro* method to evaluate the free radical neutralization capacity of phytochemical extracts. In our study, APS demonstrated significant DPPH scavenging activity, which directly reflects its electron-donating ability and radical-quenching potential. Although *in vitro* assays like DPPH do not replicate the full complexity of biological systems, they provide foundational biochemical evidence of antioxidant capacity. These findings are further supported by *in vivo* results, including significant reductions in MDA (a marker of lipid peroxidation) and increased activity of catalase (CAT) and lactate dehydrogenase (LDH), which are critical endogenous antioxidant enzymes. The observed improvements in testicular histology and sperm parameters further support the physiological relevance of this antioxidant protection.

Moreover, APS-mediated upregulation of *Nrf2* and *SOD* gene expression in testicular tissue highlights the mechanistic alignment between its antioxidant potential (demonstrated *in vitro*) and its gene-regulatory effects *in vivo*, as a previous study reported the upregulation of the *SOD* gene in relation to infertility (40). *Nrf2* is a central transcription factor that controls cellular redox balance, and its activation suggests that APS supports endogenous antioxidant defense mechanisms in addition to direct radical scavenging. In summary, the DPPH assay confirms the intrinsic antioxidant properties of APS, while the *in vivo* outcomes demonstrate systemic and cellular protection from oxidative stress and DBP-induced damage. Together, these results provide a robust and coherent understanding of APS’s protective effect through both chemical and biological antioxidant pathways. This integrated approach has been widely adopted in phytochemical antioxidant research, as combining *in vitro* radical-scavenging assays with *in vivo* biochemical and molecular endpoints offers a more comprehensive evaluation of biological efficacy (41). Furthermore, previous studies specifically on Astragalus polysaccharides have demonstrated similar antioxidant and testicular protective effects through the Nrf2 pathway (42), thereby supporting our findings.

This study has provided important insights into the mitigative role of APS against DBP-induced testicular damage in rats. These outcomes represent a positive step forward. However, this study had limitations such as a small sample size, absence of dose–response analysis, and exclusion of behavioral or fertility endpoints. It is also necessary to investigate additional genes and proteins associated with the pathway under research to verify that APS has a role in its regulation.

## 5 Conclusion

This study demonstrates that Astragalus polysaccharides (APS) effectively mitigate the reproductive toxicity induced by DBP through their antioxidative, anti-apoptotic, and protective actions on testicular tissues. APS improved semen parameters, serum testosterone levels, and testicular antioxidant enzyme activities while reducing oxidative stress markers and apoptosis-related gene expression. The modulation of the PI3K/Akt/mTOR pathway further underscores the therapeutic potential of APS in counteracting DBP-induced damage. Histopathological findings revealed significant restoration of testicular architecture in APS-treated groups. Collectively, APS show promise as a natural remedy to combat environmental toxin-induced male infertility, warranting further investigation into its clinical applications.

## Data Availability

The original contributions presented in the study are included in the article/supplementary material, further inquiries can be directed to the corresponding author/s.
